# *Eucalyptus globulus* Mediated Green Synthesis of Environmentally Benign Metal Based Nanostructures: A Review

**DOI:** 10.3390/nano13132019

**Published:** 2023-07-06

**Authors:** Muhammad Usman Sadiq, Afzal Shah, Abdul Haleem, Syed Mujtaba Shah, Iltaf Shah

**Affiliations:** 1 Department of Chemistry, Quaid-i-Azam University, Islamabad 45320, Pakistan; musmansadiq@chem.qau.edu.pk (M.U.S.); s.mujtaba@qau.edu.pk (S.M.S.); 2 School of Chemistry and Chemical Engineering, Jiangsu University, Zhenjiang 212013, China; haleem@mail.ustc.edu.cn; 3 Department of Chemistry, College of Science, United Arab Emirates University, Al Ain P.O. Box 15551, United Arab Emirates

**Keywords:** *Eucalyptus globulus*, phytonanofactory, nanostructures, green synthesis, essential oils

## Abstract

The progress in nanotechnology has effectively tackled and overcome numerous global issues, including climate change, environmental contamination, and various lethal diseases. The nanostructures being a vital part of nanotechnology have been synthesized employing different physicochemical methods. However, these methods are expensive, polluting, eco-unfriendly, and produce toxic byproducts. Green chemistry having exceptional attributes, such as cost-effectiveness, non-toxicity, higher stability, environment friendliness, ability to control size and shape, and superior performance, has emerged as a promising alternative to address the drawbacks of conventional approaches. Plant extracts are recognized as the best option for the biosynthesis of nanoparticles due to adherence to the environmentally benign route and sustainability agenda 2030 of the United Nations. In recent decades, phytosynthesized nanoparticles have gained much attention for different scientific applications. *Eucalyptus globulus* (blue gum) is an evergreen plant belonging to the family Myrtaceae, which is the targeted point of this review article. Herein, we mainly focus on the fabrication of nanoparticles, such as zinc oxide, copper oxide, iron oxide, lanthanum oxide, titanium dioxide, magnesium oxide, lead oxide, nickel oxide, gold, silver, and zirconium oxide, by utilizing *Eucalyptus globulus* extract and its essential oils. This review article aims to provide an overview of the synthesis, characterization results, and biomedical applications of nanoparticles synthesized using *Eucalyptus globulus*. The present study will be a better contribution to the readers and the students of environmental research.

## 1. Introduction

The last two decades witnessed remarkable utilization of nanotechnology in every avenue of science and technology, which is currently regarded as one of the top and demanding research areas for the safety of the society and the environment. Nanotechnology is the manipulated reorganization and restructuring of materials at the nanoscale [1]. The physicochemical characteristics of nanoscale materials markedly differ from macroscale materials due to quantum effects, enhancement of the surface area, and dominance of interfacial processes [2,3]. Albeit nanotechnology flourished exponentially after the industrial revolution, the history of human exposure to nanomaterial can be traced back to the medieval ages when gold nanoparticles were used to color glass [4]. The term “nanometer” was initially proposed by Nobel Laureate Richard Zsigmondy in 1925. He invented the word “nanometer” to define particle size and was the first to use the microscope to quantify the size of particles like gold colloids [5]. Richard Feynman explicitly can be regarded as the father of nanotechnology, which proposed the hypothesis of manipulation of atoms and molecules to generate nanoscale materials during his revolutionary lecture entitled “There is plenty of room at the bottom” at the American Physical Society 1959 meeting. Although the term nanotechnology was first used by Norio Taniguchi in a publication in 1974, and Eric Drexler popularized this term to the world in his book “Engineer of Creation” in 1986 [6].

**Figure 1:**
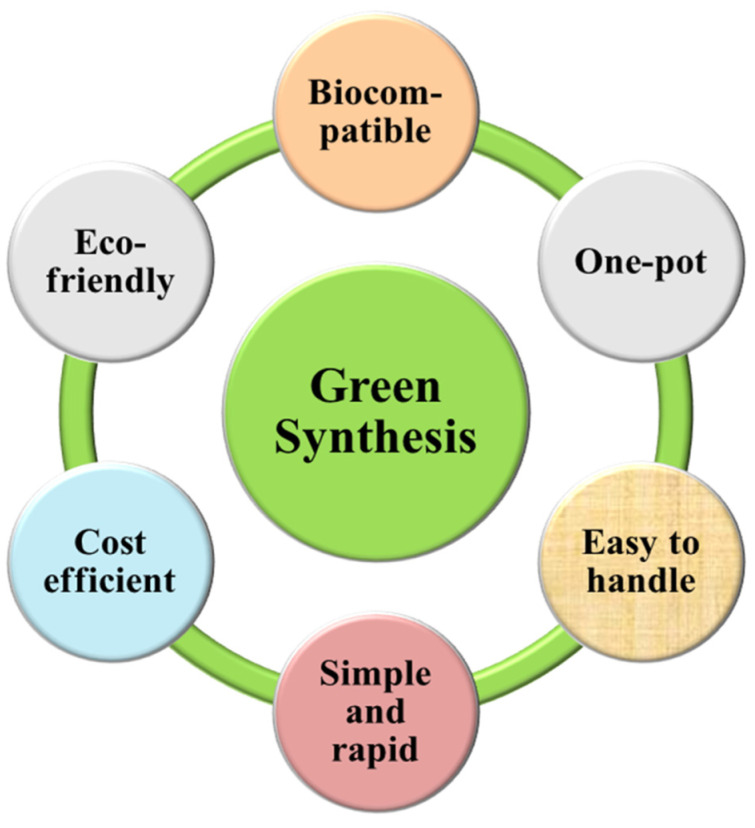
Advantages of green synthesis of nanoparticles.

To date, a considerable number of nanostructures with a variety of applications in various fields have been prepared and reported by employing different physicochemical methods (thermal decomposition, chemical reduction, microwave synthesis, ion sputtering methods, and hydrothermal methods, among others). Nanomaterials synthesized using these methods have a broad range of applications, but all these mentioned methods are not environmentally benign due to the use of bio-hazardous chemicals, which limit their uses for biomedical and clinical applications. The use of hazardous and expensive chemicals has urged scientists to exploit a cost-effective and eco-friendly synthesis approach of nanomaterials that can be an efficient alternative to these conventional methods [7]. In this regard, green methods have emerged as a potent alternative because they are easy to handle, cheaper, and environmentally safe in comparison to physical and chemical methods. The fabrication of nanostructures based on biological sources can be broadly divided into two categories: Biotemplated forming synthesis and biological synthesis. In the biotemplating method, inorganic materials are incorporated on the substrate, followed by mineralization/fossilization through various physical or chemical deposition processes, which results in the fabrication of ordered nanostructures, whereas biological synthesis relies on the metabolites of materials that result in the production of randomly distributed nanomaterials [8,9]. Albeit arguably, large-scale production of nanostructure using the green method is a difficult task, in the future, with advances in studying the composition of biological extract and the nature of reaction with metal, large-scale production will be feasible [10]. The nanomaterials fabricated employing green methods have wide applications in various fields, such as contaminant remediation, antifungal, antibacterial, biomedical, and photocatalytic activity [11,12]. Green synthesis have multiple advantagous as mentioned in Figure 1. 

Until now, plants, algae, fungi, and bacteria have been the most widely used substrates/ bio templates that play the role of bio-reductants as well as stabilizing agents owing to the reductive abilities of metabolites of these organisms [13,14,15,16].

The present review encompasses a detailed discussion of synthesis, characterizations, and applications of nanostructures synthesized using *Eucalyptus globulus* (EG) leaves, essential oils, and bark. Prior to an in-depth description of nanoparticles (NPs) synthesis, characterizations, and results, a brief overview of the physical description and phytochemicals present in EG plants is carried out.

## 2. Plant Extract-Mediated Synthesis and Characterization of Nanoparticles

A significant fraction of plants have been used in the synthesis of nanomaterials due to the reductive abilities of phytochemicals. Plants-assisted synthesis has attracted much attention as compared to other organisms due to the stable, faster, and large-scale production of nanomaterials [17]. Additionally, plants are accessible, easy to cultivate, and safe to handle. Various parts of plants (roots, fruits, peels, flowers, stems, and leaves) have been employed for the production of nanomaterials. However, leaf extracts are widely employed due to metabolite richness [18,19]. A new avenue of NPs manufacturing has emerged as a result of recent research on the biosynthesis of nanomaterials utilizing plant extracts.

**Figure 2:**
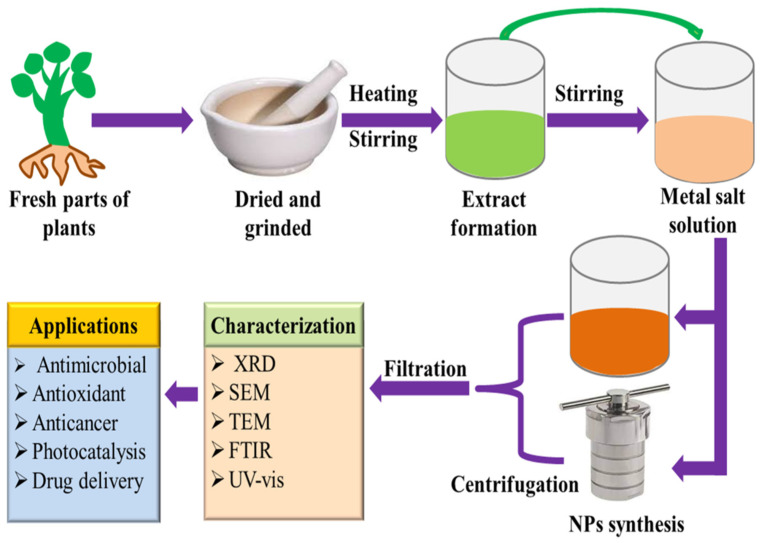
Proposed pathway of plant-assisted green synthesis of nanoparticles.

Although phytochemical-mediated synthesis of NPs has been widely adopted by the scientific community around the globe, there is still no justified reason for the selection of a particular plant during their work. It can be speculated that the facile availability, traditional and herbal uses of plants are the determining factors for their choice. Since polyphenols, alkaloids, flavonoids, saponins, polysaccharides, and other plant compounds are excellent stabilizing and reducing agents, using plant materials abundant in these compounds increases the probability of success. In the plant-assisted fabrication of nanoparticles, the salt solution and extract are simply mixed at room temperature or slightly high temperature, resulting in the synthesis of NPs within minutes. The synthesis of NPs can be initially confirmed by observing the change in the color of the solution. A detailed schematic of NPs synthesis is presented in Figure 2. 

Plant extract is mostly prepared by dissolving leaves, flowers, roots, bark, etc., in a solvent (most commonly used water and ethanol), followed by heating at low temperatures. After that, the mixture is filtered, and the filtrate is used for the synthesis of NPs. For the synthesis of NPs, the plant extract is added to the salt solution of the respective metal, followed by heating. The metal salts in the solution dissociate into metal ions. These metal ions and phytochemicals are attracted by electrostatic interaction to form a metal-phytochemicals complex, from which metal oxide NPs can be fabricated by the high-temperature calcination treatment. However, in the case of metal NPs synthesis, the biomolecules present in plant extract reduce the metal ions to the metal atoms (M°), and nucleation of M° occurs. The small-size NPs combine to form larger particles which increase the thermodynamic stability. Biomolecules present in plant extracts play a role in stabilizing and reducing the metal ions in the solution. The plant extracts contain a wide variety of phytochemicals. It is difficult to determine the precise stabilizing and reducing agents for the formation of NPs [20]. Since the phytochemicals vary from one plant to another, as well as their locality, therefore, the nature and concentration of extract, pH, the concentration of salt, contact time, and temperature are credited to influence the rate of synthesis, quantity, and other properties of NPs. Vast research on plant extract-mediated fabrication of nanostructures has been reported. The green synthesis of silver nanostructures involves the reduction of a silver metal solution to nanoscale using different metabolites present in plant extracts. The variation from light yellow to brownish color solution is a visual indicator of NPs synthesis [21,22,23]. Stable gold NPs (Au NPs) with different morphologies have been synthesized using plant extracts. Green synthesized Au NPs have catalytic and biomedical applications [24,25]. The plant extract-mediated approach has been mostly employed to prepare an enormous number of metal oxide nanostructures that have applications such as the biomedical (anticancer, antioxidant, antibacterial, antifungal, etc.), agricultural, catalytic, contaminants mitigation, and energy sectors [26].

### Characterization of Nanoparticles

The size and morphology (shape) of NPs are the most important parameters that substantially influence their physiochemical characteristics and applications [27]. These two parameters can be carefully regulated during the synthesis of NPs. The other factors which can also influence the characteristics of NPs are solubility, surface charges, surface area, porosity, zeta potential, particle aggregation, etc. Several electronic and spectroscopic techniques are utilized to evaluate the different properties (electronic, optical, mechanical, morphological, etc.) of NPs. The most widely employed techniques for the characterizations of NPs are precisely described below.

X-ray crystallography or diffraction (XRD) is employed to study the structural aspects (crystallinity, particle size, lattice dimension) of NPs. The full-width half maximum (FWHM) values of diffraction peaks are utilized to deduce the concerned information from the XRD spectrum. The diffraction peaks correspond to distinct crystallographic orientations of crystallites in NPs, thus FWHM is affected by the atoms participating in scattering events, and the contributing atoms are related to the size of the crystal plane that produces the particular reflection. The Debye–Scherrer equation is used to determine the mean crystallite size of NPs [28].

(1) $$D=\frac{K\lambda }{\beta cos\theta }$$

where D is the average crystallite size, λ is the X-rays wavelength, K is the crystallite shape factor, θ is the diffraction angle, and β is the FWHM. The crystalline structure of NPs can be estimated by comparing the intensity and, particularly, the position of peaks with the standard patterns established by the International Center of Diffraction Data (ICDD). Despite its wide utilization, this technique is not appropriate for amorphous materials and also for particles having a size less than 3 nm [29].

Scanning electron microscopy (SEM) is a versatile technique to study the surface morphology, electrical behavior, and chemical composition (SEM coupled energy dispersive X-rays analysis) of NPs. When a beam of the electron is directed on the surface of the sample (NPs), secondary electrons are emitted, which are then detected by the SEM to generate an image of the sample [29]. Contrary to SEM, transmission electron microscopy (TEM) uses the transmitted electrons to generate images of NPs. TEM can analyze the crystal structure, shape, and size at a single particle level with a capability of 200-million-fold magnification [30]. Fourier transform infrared spectroscopy (FTIR) is an environment-friendly and non-destructive technique used to determine functional groups based on how materials respond to infrared (IR) light. The molecules to be analyzed by FTIR spectroscopy must have a dipole moment. The FTIR analysis of NPs synthesized using plant extracts is mostly used to carry out to investigate the phytochemical makeup of extracts that act as capping agents [31]. UV-visible spectroscopy (UV-Vis) is widely employed to investigate the optical properties (absorption and band gap) of NPs. The absorption pattern obtained using UV-Vis spectroscopy is used to characterize the NPs. Furthermore, the band gap is used to analyze the photocatalytic behavior of NPs. Thermogravimetric analysis (TGA) is employed to study the thermal stability of nanostructures. X-ray photoelectron spectroscopy (XPS) is employed to determine the purity of NPs by analyzing the binding energy peaks and chemical states of the materials. Typically, XPS can probe the sample up to 10 nm [32]. Furthermore, Brunauer–Emmett–Teller (BET) study is employed to investigate the porosity of NPs.

## 3. Nanostructures Phytosynthesized Using *Eucalyptus globulus* Extract and Essential Oils

The Myrtaceae family, which consists of 140 groups and approximately 3800 species, is found throughout the world’s subtropical and tropical regions [33]. Eucalyptus is one of the most significant genera of this family, which is extensively cultivated in the world. *Eucalyptus globulus* (EG) is an endemic plant in Australia that is widely found throughout the world [34]. It is the main source of botanical essential oils and is well-recognized in pharmacopeia around the globe. These essential oils have broad applications as astringents, anesthetics, deodorants, antiseptics, diaphoretics, febrifuges, disinfectants, expectorants, preventatives, insect repellents, sedative inhalants, as well as in diabetes, cancer, bronchitis, flu, fever, diphtheria, diarrhea, dysentery, worm, sore throat, rhinitis, and wound treatments. These are also used in the cosmetic and soap industries [35].

EG plants are included among the world’s tallest tree and grow very quickly. The bark is ash grey and sheds annually in the form of long ribbons or flakes when the underneath layer develops. The leaves have leathery textures that frequently hang vertically or obliquely and contain aromatic oils in circular glands [36,37]. An operculum is formed by the fusion of petals and sepals that enclose the stamen. This trait is found in the whole genus, and from this trait, the characteristic name, eucalyptus, originated. The term eucalyptus comes from the Greek words “eu” and “kalyptos”, which mean “well” and “covered”, respectively [38].

With regard to extensive and conventional uses, numerous secondary metabolites, including oxygenated sesquiterpenes, oxygenated monoterpenes, monoterpenes, and sesquiterpenes, have been extracted from EG leaves and structurally explained worldwide [39,40]. Eucalyptol (1, 8-cineole) is a key constituent present in nearly all the essential oils (EOs) of this genus that are grown in various regions around the globe. EOs are complex liquid mixtures of low molecular weight volatile compounds extracted from aromatic plants obtained by using hydrodistillation, steam distillation, or solvent extraction [41]. However, the percentage of eucalyptol varies from one region to another. It has been reported that the eucalyptol content of EG leaf oil varies as follows: 84.5% in Iran [42], 64.5% in Uruguay [43], 77% in Cuba [44], 86.7% in California, and 50–65% in Argentina [45], 90% in Australia [46], 44.4% in India [47], 86.5% in Indonesia [48], and 85.5% in Montenegro [49]. Geographical and climatic factors have been identified as causes contributing causes to these differences. Other main constituents of EOs are α-pinene, β-cymene, p-cymene, o-cymene, α-phellandrene, and iso-valeradehyd [50]. The EOs of EG have also been found to be a potential candidate of biological importance (anti-quorum, antimicrobial, and antioxidant potential) [51].

EG plants are rich in phytochemicals and their biomedical importance has been scientifically proven, which is shown in Table 1.

According to Said et al. [40], the EOs of EG exhibit satisfactory activity against *S. aureus* and minimal activity against *E. coli*. The presence of 1,8-cineole, globulol, and aromadendrene in the EOs may be responsible for their antibacterial effect. Aromadendrene has lipophilic nature, which allows it to cross the cell membrane. Additionally, it has a cyclopropanone ring and methylene moiety which alkylate to the protein and disturb their conformations [67]. The EOs of EG exhibit excellent antioxidant activity through their ability to scavenge DPPH free radicals. This may be ascribed to the presence of 1, 8 cineole and the synergistic impact of other components of EOs to scavenge the free radicals [39,60]. EOs of EG demonstrated excellent antifungal efficiency against *Candida albicans* (*C. albicans*). Eucalyptus surpassed nystatin (an antifungal drug used to treat fungal infections of the mouth, vagina, skin, and intestinal tract) by a factor of two in its capacity to attack *C. albicans* due to the high concentration of 1,8-cineole in the EOs [64].

### 3.1. Zinc Oxide Nanoparticles (ZnO NPs)

**Figure 3:**
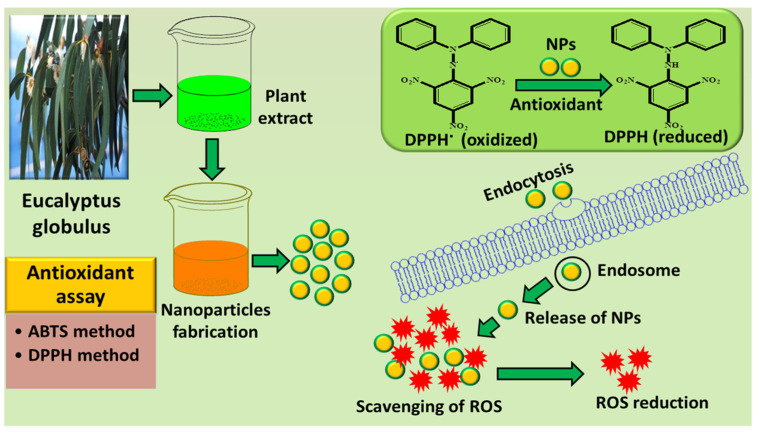
Antioxidant activity of EG extract-mediated NPs.

ZnO NPs possess indispensable biological, chemical, and physical characteristics, particularly, environmental friendliness, biocompatibility, innocuous nature, and low cost, which make them a suitable choice in material sciences [68,69]. It is widely utilized in various environmental, biological, industrial, and energy applications due to its distinctive properties [70,71,72,73]. Considering these characteristics and applications, Siripireddy and Mandal [74] were the first to use the leaves of EG to synthesize ZnO NPs. They fabricated ZnO NPs by mixing equal volume (20 mL) of EG extract and 0.1 N zinc nitrate hexahydrate aqueous solution. The reaction was stopped after the appearance of brown-colored precipitates and kept undisturbed for 24 h. The precipitates were centrifuged and washed multiple times with ethanol and dried in an oven at 80 °C, followed by annealing at 400 °C for 2 h. Different morphological aspects of ZnO NPs were investigated using various analytical techniques such as Field emission scanning electron microscopy (FE-SEM), TEM, Energy dispersive X-ray spectroscopy (EDX), XRD, UV-Vis, dynamic light scattering, and FTIR spectroscopy. The adsorption and photocatalytic activity of ZnO NPs were explored utilizing methyl orange (MO) and methylene blue (MB) dyes. Both dyes were removed only up to 12% by adsorbing on the surface of ZnO NPs. The photocatalytic degradation of dyes under a UV light source showed that MB was degraded up to 98.82%, whereas MO was degraded up to 96.6% within the same time span as for the adsorption study. The DPPH scavenging assay was used to investigate the antioxidant activity of ZnO NPs, which demonstrated a scavenging capability of up to 82%, with an IC_50_ value of 46.62% µg/mL. The smaller particle size and transfer of electron density from the oxygen atom of ZnO to the nitrogen atom of DPPH are responsible for the antioxidant activity. Free radicals are highly unstable atoms, ions, or molecules with unpaired electrons that are extremely reactive to other molecules. Sulfur, oxygen, and nitrogen are the most common source of free radicals [75]. Reactive oxygen species (ROS) are free radicals of oxygen species that comprise hydroxyl radical (**^.^**OH), hydroperoxyl radical (HO_2_**^.^**), and peroxide ion (O_2_^−^). In the human body, free radicals are produced as normal metabolic products, but they have harmful effects. If there is a balance between ROS and antioxidants, then the body cells will perform their normal functions. On the other hand, if this balance is disturbed, then cells have to endure the oxidative stress of ROS. Antioxidants can be defined as “any natural or synthetic substance that can prevent, remove, or delay oxidative damage to the substrate when present in smaller quantity in comparison with the substrate” [76]. NPs can act as antioxidants by either hydrogen transfer or electron transfer. In the electron transfer process, electron donation from antioxidant species results in the reduction of oxidative molecules, whereas in hydrogen transfer, scavenging of free radical occurs by hydrogen atoms [77]. The general mechanism of antioxidant activity of EG extract-mediated NPs is illustrated in Figure 3.

The most widely adopted methods to evaluate the antioxidant activity of NPs are DPPH and ABTS methods. Green synthesized NPs show enhanced antioxidant activity due to the presence of phytochemicals on the surface of NPs. Polyphenols, such as 1, 8 cineole and thymol, are the major constituents of EG phytochemicals, which are responsible for the enhanced activity of EG-mediated NPs [78]. Antioxidant activity of metal oxide NPs (such as ZnO, CuO, etc.) synthesized using EG extract is due to the transfer of electrons from the oxygen of NPs to unpaired electrons on the nitrogen atom of DPPH free radicals, which scavenge the free radical resulting in the formation of stable DPPH molecule. The electron-hole pairs formed on the surface of NPs generate a high redox potential which causes water to split into hydrogen and hydroxyl radicals. These radicals cause the reduction of DPPH radicals and result in the formation of stable DPPH molecules [74].

Shahid et al. [78] screened the antibacterial, antioxidant, and anticancer activity of ZnO NPs synthesized by utilizing EG leaf extract and by using a chemical method. The biosynthesized ZnO NPs were characterized using XRD and TEM analysis, which confirmed the synthesis of spherical NPs with an average size of 12.64 nm. The antibacterial potential of ZnO NPs against *Klebsiella pneumoniae* (*K. leb*), *Escherichia coli* (*E. coli*), *Staphylococcus aureus* (*S. aureus*), and *Streptococcus pseudopneumoniae* (*S. pseudo*) was analyzed using disc diffusion method by using Gentamycin as a control. The EG extract-mediated ZnO NPs showed higher activity in comparison to chemically synthesized NPs. The activity was ascribed to the phytochemicals attached to ZnO NPs, and the order of reactivity was found to be *S. pseudo* > *S. aureus* > *K. leb* > *E. coli.* The antioxidant potential of ZnO NPs was tested by using the DPPH method. The findings revealed the enhanced antioxidant activity of EG-mediated ZnO NPs relative to NPs produced by the chemical method at all concentrations, which can be dedicated to the small size of NPs and phenolic compounds present in EG extract. MMT assay was used to investigate the anticancer activity of EG-mediated ZnO NPs in breast cancer line cells. The results demonstrated the magnificent performance of ZnO NPs at various concentrations. 120 µg/mL concentration of ZnO NPs showed maximum anticancer activity having cell viability comparable to control.

Further, Barzinjy and Azeez [79] used the EG leaf extract and zinc nitrate hexahydrate for the creation of ZnO NPs with slight modification. For NPs fabrication, 30 mL of leaf extract was heated on a hotplate when the extract attained 60 °C, then 3 g of the metal precursor was added to the extract solution. After washing with ethanol and distilled water, the powder was dried at 100 °C. The synthesis of pure hexagonal-shaped ZnO NPs having an average diameter of 39 nm was confirmed using FESM and EDX analysis. The UV-Vis spectrum depicted an absorption maximum at 375 nm, which is the characteristic peak of ZnO NPs. XRD results revealed the hexagonal wurtzite form with an average crystallite size of 27 nm and 99.94% crystallinity. Other characterization techniques include differential scanning calorimetry (DSC), TGA, FTIR, and dynamic light scattering (DLS).

Further, scientists investigated the antifungal activity of ZnO NPs synthesized using EG leaf extract as a reducing agent and zinc nitrate hexahydrate as a metal precursor [80]. The synthesis route involved the mixing of a 1 nM solution of zinc nitrate hexahydrate and leaf extract in a ratio of 1:2 with continuous stirring at 150 °C for three hours. The resultant material was centrifuged, followed by washing and drying at 80 °C for 5–6 h. On instrumental analysis, FTIR spectra showed the presence of essential phytochemicals of EG, like carvacrol, borneol, camphene, citronellal, etc., which act as stabilizing agents. UV-Vis result showed an absorption peak at 300 nm, while SEM examination revealed that the majority of the ZnO NPs were spherical, with a small number of curved particles. The antifungal activity of these NPs was assessed against *Alternaria mali* (*A. mali*), *Diplodia seriata* (*D. seriata*), and *Botryosphaeria dothidea* (*B. dothidea*) fungal species having harmful effects on apple orchards. ZnO NPs demonstrated 55.2%, 65.4%, and 76.7% growth rate suppression against *D. seriata*, *B. dothidea*, and *A. mali*, respectively, at 100 ppm concentration. The SEM examination confirmed the microscopic findings of fungal growth exposed to ZnO NPs, which demonstrated that NPs damage fungal hyphae’s surface and trigger the release of cellular components, which results in the shrinkage of hyphae.

Obeizi et al. [81] published a study on the preparation of ZnO NPs utilizing EG leaves oil. Before ZnO synthesis, the extraction of essential oil was performed using the hydrodistillation method. Three mL of essential oil was produced by hydro-distilling 300 g of fresh EG leaves using a Clevenger apparatus, which was analyzed using Gas-Chromatography-Mass spectrometry. For ZnO NPs preparation, EG oil was diluted using acetone. Five mL of diluted EG oil was added to boiling 30 mL of 0.1 mM zinc acetate dihydrate at pH 7, which resulted in the color change from visible to light yellow, indicating the formation of ZnO NPs. Centrifugation, washing, and drying, followed by calcination at 550 °C resulted in the formation of powder ZnO NPs. SEM micrographs showed needle-like and spherical morphology formation, whereas EDX analysis showed the characteristic peaks of O and Zn without any impurity. The average crystallite size of 24 nm was computed using XRD results. Other analytical techniques, such as UV-Vis, FTIR, and DLS further supported the fabrication of nanometer-sized ZnO NPs. The biomedical potential was assessed using antimicrobial and anti-biofilm assays. Antimicrobial activity was evaluated against ten different bacterial species. The lowest minimum inhibitory concentration (0.5 µg/mL) for *Candida albicans* showed the efficacy of ZnO NPs. ZnO NPs’ anti-biofilm activity was assessed using a 96-well microtiter plate technique against *S. aureus* and *Pseudomonas aeruginosa (P. aeruginosa).* The findings demonstrated that ZnO NPs effectively and dose-dependently way inhibited the biofilm that is generated by the two bacterial strains. A significant inhibition rate (85–97%) is seen for the two bacteria at a dose of 100 g/mL ZnO NPs.

Razanamahandry et al. [82] biosynthesized ZnO NPs by heating aqueous EG leaf extract and different concentrations of zinc nitrate hexahydrate at 45 ℃. The resultant mixture was heated at 60 °C to obtain the precipitates. In an open furnace, the dried powder underwent a two-hour annealing process at 500 °C. The morphological study confirmed the formation of spherical NPs at all concentrations. Aside from the signals of Zn and O in the EDX result, sodium, calcium, sulfur, and phosphorous were also detected, which was attributed to extract, and the signal of carbon was attributed to the use of a carbon coat during SEM examination. XRD analysis revealed an increase in the crystallite size at higher concentrations of salt solution, whereas all samples were found in the hexagonal wurtzite phase. All produced ZnO NPs showed emission photoluminescence peaks at 480 nm, which revealed the presence of defects. Zn-O bending at 758 cm^−1^ in FTIR spectra indicated the formation of ZnO NPs. The potential of ZnO to eliminate FCN from wastewater by photocatalysis was investigated under UV and solar light irradiation. Under UV light exposure, 3 g of CN^−^ L^−^ degraded up to 98% in 20 min, while under solar light exposure, 45% degradation was observed.

To explore biological utilities Masood and co-workers fabricated ZnO NPs using EG extract [83]. XRD, UV-Vis, and FTIR studies were employed to characterize ZnO NPs, affirming their fabrication, size, and structure. The absorption peak at 360 nm indicated the ZnO NPs fabrication, while the XRD results demonstrated the formation of hexagonal ZnO NPs having a 36 nm average diameter. The antimicrobial effects of EG leaf extract, ZnO NPs, and rhizobacteria against *E. coli* were evaluated utilizing disc diffusion and well diffusion strategies. An in-silico strategy was used to find the prediction model for virtual screening of how NPs are used to regulate Colibacillosis by forecasting how ZnO NPs (ligands) interact with the receptor of *E. coli*. EG extract and ZnO NPs both showed appreciable antimicrobial activity, which increased by increasing concentration and incubation time. The antibacterial activity tests showed that the synergistic use of rhizobacteria and ZnO NPs considerably decreased the bacterial pathogen *E. coli.* The antagonism’s method of action is through ligand-receptor binding, according to in silico research.

Recently, Siddique et al. [84] reported the ZnO NPs fabrication for the evaluation of an insecticidal study against *Rhyzopertha Dominica* (*R. dominica*). Briefly, 4 g of zinc precursor was added to boiling EG extract (70–80 °C), followed by heating for 2 h at 400 °C. SEM results demonstrated the formation of spherical, walnut-shaped, and flower ZnO NPs. UV-Vis spectrum showed the characteristic peak of ZnO at 370 nm, which indicated the successful preparation of ZnO NPs. Insecticide assays were carried out using six concentrations of EG extract and EG-mediated ZnO NPs, respectively, and their dosage rate was examined after four exposure durations. After 15 days of exposure, insect mortality was 62.5% due to EG leaf extract at 1800 ppm dose rate with LC_50_ value of 1043.06 and 80.5% due to ZnO NPs at 600 ppm dosage rate with LC_50_ value of 202.11 ppm respectively. After 30 days, *R. dominica* growth inhibition was 75.7% against EG extract and 87.0% against ZnO NPs. These findings showed that EG leaf extract and ZnO NPs can be utilized as an environment-friendly strategy for pest management in stored products.

**Figure 4:**
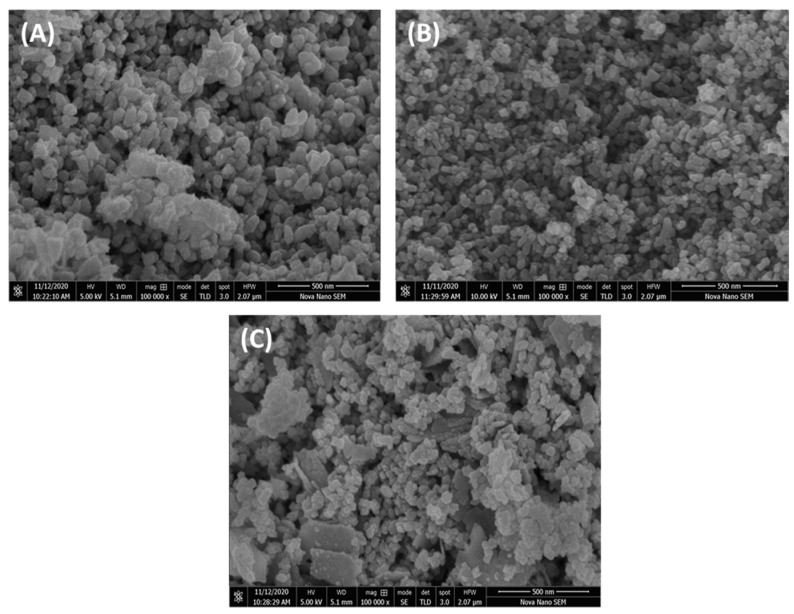
SEM images of (**A**) ZnO NPs, (**B**) CuO NPs, (**C**) ZnO/CuO nanocomposite. Reprinted from Ref. [85].

The EG extract-mediated fabrication of ZnO NPs, copper oxide nanoparticles (CuO NPs), and ZnO/CuO nanocomposite having a size less than 30 nm was claimed and was evaluated for the photodegradation of MO [85]. The SEM micrographs of all three samples showed the agglomeration of particles, which was related to the hydrolysis and heat treatment. The ZnO NPs (55.41 nm) showed irregular morphology having clear boundaries (Figure 4A), whereas CuO NPs (36.28 nm) were spherical and regular in shape (Figure 4B). The ZnO NPs in ZnO/CuO nanocomposite (39.19 nm) were in the form of aggregated chunks on which the CuO NPs were distributed (Figure 4C). The EDX results demonstrated the fabrication of highly pure NPs and nanocomposite. A trace amount of carbon was also detected in all samples, which was related to utilization of plant extract. The production of hexagonal ZnO and monoclinic CuO NPs having 28.3 nm and 42.8 nm crystallite sizes were determined from the XRD study. The XRD of the ZnO/CuO composite showed that ZnO was hexagonal, whereas CuO was in a monoclinic crystalline shape. A reduction in band gap energy was observed for the ZnO/CuO (1.48 eV) in comparison to ZnO (3.36 eV) and CuO (1.83 eV) NPs from DRS results. The FTIR finding demonstrated the effective capping role of EG extract for the engineering of stable NPs and nanocomposite. The photodegradation of methyl orange was performed by varying the dose of catalyst (0.02–0.06 g) and pH values of 7 and 12. A decrease in degradation was observed by increasing the dose of catalyst. The optimum degradation was observed at 0.02 g amount of catalyst and pH 12. A comparison of the size and morphology of ZnO NPs synthesized using EG leaf extract and essential oils can be seen in Table 2. ZnO NPs having spherical morphology was prepared in the majority of cases, however, variation in size is most prominent. The variation and size and morphology of ZnO NPs can be related to the phytochemical variation with regional variation and might be due to the different experimental conditions used for the synthesis of ZnO NPs.

### 3.2. Copper Oxide Nanoparticles

CuO NPs is a p-type semiconducting material possessing a direct band gap (1.21–2.15 eV) and monoclinic structure [86,87]. The potential utility of CuO NPs in photovoltaics, food preservation, catalysis, superconductors, dye elimination, wastewater treatments, and biological fields has been recognized [88,89,90]. To further increase the realm of the environmentally benign approaches of CuO NPs synthesis, Pinto et al. [91] adopted an eco-friendly route for the fabrication of CuO nanowires utilizing EG bark extract as stabilizing/reducing agent and oleylamine and oleic acid as surfactants. In a typical synthesis, 34.1 mg of dihydrated copper chloride was dissolved in distilled water. In a different beaker, oleylamine and oleic acid were added to ethanol while being magnetically stirred. The ethanolic solution was added dropwise to the CuCl_2_.2H_2_O solution in a flask to which distilled water was also added. To facilitate the synthesis of copper-oleylamine precursor, the mixture was stirred magnetically for 12 h at 50 °C. After adding EG extract drop-by-drop to the mixture, it was transferred to an autoclave, which was then kept at 121 °C for 2 h. Copper nanowires were then centrifuged, followed by sonication and washing. UV-Vis spectra showed a shift in absorbance to a higher value (584 to 613 nm) with increasing extract concentration, which suggests the formation of longer nanowires at higher extract concentration. FTIR spectra revealed the C-H stretching bands, which were ascribed to the attachment of oleyl groups to nanowires. TEM and SEM results demonstrated the formation of nanowires along with a negligible amount of nanocubes, nanoprisms, and nanosphere. The average diameter of nanowires increased with increasing extract concentration. These findings were in accordance with UV-Vis results. According to the XPS analysis, the nanowires’ surface has a significant quantity of C, O, and Cu. These results showed that the sugars and phenolics that were initially present in the extract adsorbed onto the surface of the copper nanowires.

Another investigation to explore the biological applications of biosynthesized CuO NPs was done by K. Ali et al. [92] using EG extract. The CuO NPs synthesis was performed by varying experimental conditions, such as EG extract and pH. The EG-mediated CuO NPs were characterized using sophisticated techniques to explore their morphological and compositional properties. GC-MS analysis of EG extract led to the identification of 17 distinct types of terpenoids. Terpineols, β-eudesmol, benzamidophenyl-4-benzoate, and 2,6-octadienal-3,7-dimethyl, were four bioactive terpenoids that were shown to be linked with the CuO NPs as capping agents and most probably responsible for nucleation and stability of EG-CuO NPs. According to flow cytometric (FCM) data, terpenoids encapsulated EG-CuO NPs have a significantly higher intracellular uptake propensity and accumulate more intracellular reactive oxygen species that kill planktonic cells of clinical isolates of the bacteria *E. coli*, *P.aeruginosa*, and *S. aureus* in comparison to commercial nano and bulk sized CuO.

In a recent study documented by Alhalili [93], CuO NPs were prepared using EG leaf extract and copper sulfate. The synthesis route involved the mixing of EG leaf extract with 1 mM copper sulfate solution in a 1:9 ratio with constant stirring for 2 h, after which the appearance of brown color indicated the NPs formation. The colloidal suspension was incubated for 24 h, followed by centrifugation and washing. The final product was calcined at 400 °C to remove any organic moiety attached to CuO NPs. XRD results revealed that CuO NPs have a monoclinic crystalline shape having crystallite size in the range of 38.571–145.15 nm. Vibration peaks at 580 and 530 cm^−1^ in FTIR spectrum affirmed the synthesis of CuO NPs. FESEM photograph showed rod-like morphology of synthesized CuO NPs. Methyl orange (MO) was utilized to evaluate the adsorption characteristics of the CuO nano-adsorbant. Various parameters, such as pH, dose effect of nano-adsorbant, and dye concentration, were optimized to get maximum adsorption of dye. The impact of pH on the adsorption efficiency of CuO NPs was investigated in the range of 2–11 pH. As pH rose to 6, MO adsorption on CuO NPs increased and then declined. Based on the outcomes of the experiments, pH 6.5 was determined as an optimum pH. The adsorption of dye on adsorbent depends on the binding sites and surface area. The dose of CuO was varied from 0.01–0.1 g with an optimum dose value of 0.04 g. Under the optimized conditions, 95 mg/g adsorption efficiency was obtained at room temperature.

### 3.3. Magnesium Oxide Nanoparticles

Nano-magnesium oxide (MgO NPs) has grabbed manifold higher attention than many other metal oxide NPs owing to its biocompatibility, cost-effective fabrication, biodegradability, stability, promising corrosion resistance, excellent thermal conductivity, optical transparency, and eco-innocuous nature [94,95,96]. The potential of Mg NPs for various biological (anticancer, antibacterial, and antioxidant), environmental (catalysis, photocatalysis, and adsorption), and electrochemical application (biosensors) have been explored [97,98,99]. Jeevanandam and colleagues reported the synthesis of EG-mediated synthesis of magnesium oxide nanorods (MgO NRs) [100]. Different experimental parameters, such as concentration of the salt solution, reaction times, and extract volume, were optimized to obtain smaller-sized MgO NRs. The concentration of precursor salt solution (magnesium nitrate hexahydrate) varied from 0.001 to 0.1 M. The size of NRs was found to rise as the concentration of the salt solution was increased. At higher salt concentrations, phytochemicals did not effectively control the size, which resulted in the size increment. The effect of EG extract on the size was studied from 3 to 7 mL, with an increase in volume, a decrease in particles’ size observed. It was attributed to an increased quantity of phytochemicals, which effectively capped the particles resulting in reduced-sized MgO NRs. Similarly, reaction time varied from 15 to 25 min. The MgO NRs obtained at a reaction time of 20 min were found to be smaller in comparison to 15 min and 25 min reaction times. To synthesize smaller-sized MgO NRs, optimum conditions, such as 7 mL of EG extract, 0.001 M of salt concentration, and 20 min at 80 °C, were chosen. A robust, broad peak at 3319.46 cm^−1^ and a narrow peak at 1974.81 cm^−1^ were observed in the FTIR spectrum of EG leaf extract, which shifted to 3316.48 cm^−1^ and 1982.02 cm^−1^ in MgO NRs, respectively. The O-H stretching vibrations that resulted in these bands could be caused by functional groups, such as ester, carboxylic acid, alcohol, and ether. The UV-Vis spectrum had two peaks at 340 and 607 nm. The peak observed at 340 nm validated the fabrication of MgO NPs, while the peak at 607 nm was ascribed to the rod-like structure. The morphological analysis by TEM demonstrated the stacked NRs having 6–9 nm width. The average size (43.82 nm) computed by DLS studies was contradictory to TEM results. This was owing to the spherical approximation of particles, which is a major fault of DLS analysis for studying rod-like structures.

### 3.4. Iron Oxide Nanoparticles

Iron oxides occur in approximately 16 different forms, but hematite (αFe_2_O_3_), maghemite (γFe_2_O_3_), and magnetite (Fe_2_O_3_) are the most prevalent forms [101]. Hematite is also recognized as kidney ore, red ochre, martite, ferric oxide, iron sesquioxide, and specularite. Finely divided hematite is red-blood in color, while coarsely crystalline is grey or black. Magnetite, which possesses the maximum magnetic abilities out of all transition metal oxides, is commonly referred to as magnetic iron ore, black iron oxide, loadstone, Hercules stone, and ferrous stone. Maghemite, on the other hand, occurs in soil as a result of magnetite weathering or heating of various iron oxides. Iron oxide nanoparticles gained significant attention owing to their appealing superparamagnetic features, low toxicity, surface area-to-volume ratio, and easy separation procedure [102,103]. Iron oxide nanoparticles (FeO NPs) were prepared by Matheswaran Balamurugan et al. [104] using EG leaf extract and iron (Ⅲ) chloride as precursor salt solution. The fabrication of FeO NPs was carried out by infusing 20 mL of EG extract to iron (Ⅲ) chloride aqueous solution while being stirred at normal atmospheric pressure and ambient temperature. The change in the solution color from yellow to greenish-black occurred within three minutes which indicated the synthesis of NPs. UV-Vis spectrum showed an absorption maximum at 402 nm, suggesting the synthesis of β-Fe_2_O_3_. SEM and TEM images showed that NPs were agglomerated, which might be done to reduce the surface energies. X-ray Diffraction (XRD) analysis was used to characterize the structural properties, which demonstrated the well-crystalline rhombohedral geometry of β-Fe_2_O_3_. A broad peak at 544 cm^−1^ was observed in the FTIR spectrum, which represented the Fe-O stretching vibrations.

**Figure 5:**
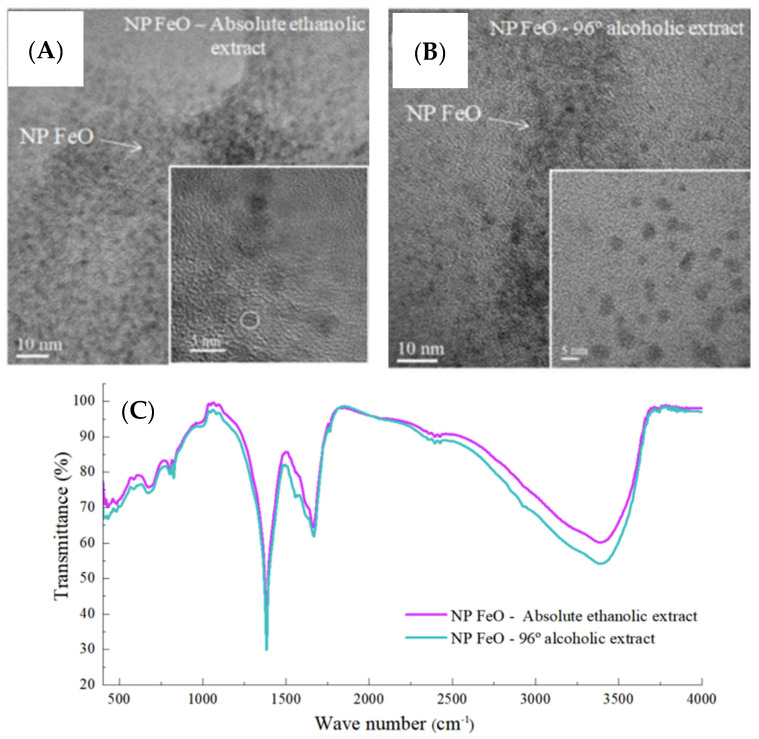
TEM micrographs of FeO NPs synthesized using (**A**) absolute ethanolic extract, (**B**) 96° alcoholic extract, (**C**) FTIR spectra of FeO NPs. Reprinted from Ref. [105].

Andrade-Zavaleta et al. [105] performed the fabrication of FeO NPs using alcoholic (absolute ethanol and alcohol 96%) EG leaf extract. Before the preparation of the extract, leaves were cleaned with ultrapure water and dried in an oven at 70 °C to remove the moisture. Leaf extracts were prepared by adding dried leaves (5 g) to a 50 mL solvent mixture at room temperature, followed by stirring for 30 min. Synthesis of FeO NPs was performed separately for both extracts by adding 15 mL of each extract to iron nitrate nonahydrate (50 mL) solutions. After the specified time, solutions were heated using a water bath to completely remove liquid content, and eventually, black residue was obtained, indicating FeO NPs. FeO NPs fabricated using 96% alcohol showed a peak at 391.2 nm, whereas FeO NPs synthesized utilizing absolute ethanol demonstrated an intense peak at 393.4 nm in the UV-Vis spectra. TEM micrographs revealed that the solvent significantly influenced the particle size of FeO NPs, although morphology remains the same for both extracts. FeO NPs synthesized using absolute ethanolic extract show spherical morphology with an average particle size of 2.34 ± 0.53 nm (Figure 5A), whereas FeO NPs fabricated using 96% ethanolic extract reveal spherical morphology with an average size of 4.17 ± 1.22 nm (Figure 5B). According to the FT-IR data, the precursor reduction process is directly impacted by the presence of aromatic compounds (Figure 5C). XRD data were used to determine the crystalline phase and crystallite size. In both cases, FeO NPs have spherical geometry with 2.863 nm (96% alcohol) and 1.715 nm (absolute ethanol extract) crystallite size. Furthermore, XRD analysis showed the existence of magnetite and maghemite magnetic phases. FeO NPs synthesized using 96% alcohol was evaluated for their potential to alleviate the heavy metals from soil, which showed outstanding potential for soil remediation. FeO NPs effectively removed heavy metals like Cr-VI, Cd, and, to a lesser degree, Pb.

### 3.5. Lead Oxide Nanoparticles

Nano lead oxide (PbO NPs) has been widely employed in lead storage batteries. PbO NPs also find usages such as electrodes, detectors for X-ryas imaging, lead crystal, lead glazes, decorative potteries, sensors, and paints [106,107,108]. PbO is a semiconducting substance found in two forms: red tetragonal (alpha form) and yellow orthorhombic (beta form) [109]. Fabrication of PbO NPs employing different physicochemical approaches has been documented. To minimize the hazardous disadvantages (toxic chemicals and toxic waste) of these methods, energy consumption, and toxicity of lead alternative, environmental benign and green approaches have been adopted to fabricate PbO nanostructures. The green methods of PbO NPs synthesis can improve their properties while decreasing the consumption of toxic chemicals utilized during their production [110]. Tailor and Lawal [111] documented a detailed study on PbO NPs synthesis using EG leaf broth and evaluation of their biological applications along with phytochemical screening. Soxhlet apparatus was used for the extraction of plant materials using different solvents till complete extraction. For the biosynthesis of PbO NPs, 0.1 M lead acetate solution (90 mL) was mixed with 10 mL of plant extract to make 100 mL total volume. The solution was incubated in dark for 24 h before being decanted to get residue. Several phytochemical analysis tests (Liebermann–Buchard test, sulfuric acid test, Shinoda test, sodium hydroxide test, alkaloids tests, ferric chloride test, frothing test, Kella–Killani test, ninhydrin test, million’s test, and test for reducing sugars) were performed to diagnose the constituents present in EG extract. The results of phytochemical screening showed the presence of glycosides, alkaloids, carbohydrates, anthraquinones, saponins, cardiac glycosides, flavonoids, phenolic compounds, steroids/terpenes, reducing sugars, and tannins, and the absence of glycosides and amino acids for different extracts of EG. SEM-based morphological examination revealed that PbO NPs were aggregated, having an average diameter of 34.61 nm. UV-Vis spectrum showed a major peak at 398 nm and a minor peak at 225 nm which were attributed to the presence of phenolic compounds in the extract. The peaks at 654.04 and 772.78 cm^−1^ in the FTIR spectrum corresponded to Pb-O stretching vibrations. The antibacterial potential of EG extracts and PbO NPs synthesized using various EG extracts were screened against strains of *E. coli* and *S. aureus*. Despite having less antibacterial power than EG extract, PbO NPs demonstrated high antibacterial capability against both bacteria.

### 3.6. Nickel Oxide Nanoparticles

**Figure 6:**
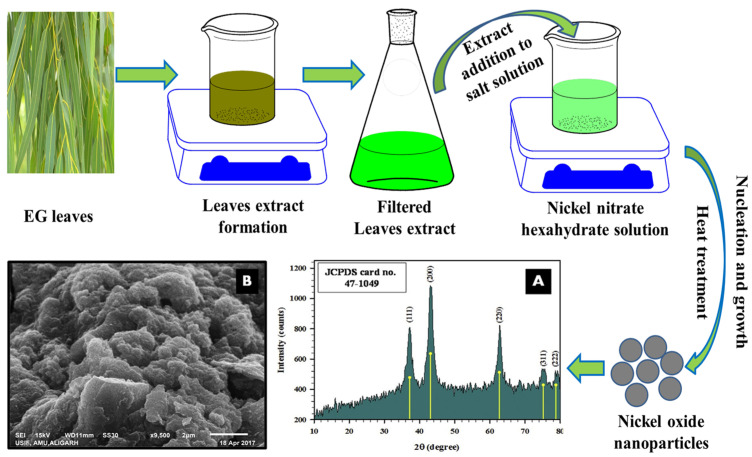
Synthesis of NiO NPs using EG extract, (**A**) XRD spectrum of NiO NPs, (**B**) SEM image of NiO NPs. Reprinted with permission from Ref. [116]. Copyright 2017 Elsevier.

Nickel oxide has captivated the attention of scientists owing to its distinctive physical, biological, chemical, and optical characteristics. It has demonstrated promising results in various fields, such as catalysts, supercapacitors, gas sensors, electrochromic devices, and biological utilities [112,113]. Over the past few years, phytochemicals-aided synthesis of NiO NPs has garnered much attention due to the fast, simple, cheapest pathway and also due to the possibility of fabrication of various morphologies [114,115]. Saleem et al. [116] evaluated the bactericidal potential of NiO NPs fabricated utilizing EG extract as capping agents and nickel nitrate hexahydrate under different experimental conditions. The EG extract was combined with a 1 mM nickel nitrate hexahydrate solution, which was then vigorously stirred at 70 °C. Centrifugation of solution was performed to obtain sediments, which were subsequently dried at 60 °C and 100 °C to get NiO NPs. Numerous spectroscopic methods, including FTIR, UV-Vis, EDX, XRD, and SEM were used to explore the characteristics of NiO NPs. UV-Vis spectra of NiO NPs synthesized under different conditions showed SPR wavelength ranging from 374 to 435 nm. SPR wavelength of NiO NPs increased with an increase in metal precursor and decreased by increasing EG extract. This change in SPR was attributed to the presence of more phytochemicals, which led to excessive NiO NPs capping, resulting in small-sized NPs, hence the blue shift in SPR. Similarly, increasing the pH value increased SPR, implying the creation of bigger particles. XRD analysis showed the formation of a face-centered cubic structure having 19 nm particle size (Figure 6A). The SEM (Figure 6B) and TEM-based morphological studies revealed the fabrication of polymorphic aggregates with an average diameter of 14 nm. Vibrations at 455 cm^−1^ in FTIR spectrum showed Ni-O bond formations. The antibacterial anti-biofilm potential of NiO NPs was tested against methicillin-resistant *S. aureus* (MRSA), *E. coli*, *P. aeruginosa*, and methicillin-sensitive *S. aureus* (MSSA). Bacterial cell growth treated with the lowest concentration of NPs was slightly sluggish than in the control group. The growth of all examined strains was constrained and nearly nonexistent at the lowest bacterial concentration levels when cells were subjected to increasing concentrations of NPs. SEM micrographs of *E. coli* and methicillin-resistant *S. aureus* treated with NiO NPs showed a significant number of gaps, disordered outer surfaces, pits, shrinkage, and fragmented membrane consistency in comparison to *P. aeruginosa* and methicillin-sensitive *S. aureus*. The antibiofilm potential of NiO NPs was assessed using glass coverslips as well as microtitre plates, with the results demonstrating the antibiofilm activity of NiO NPs.

### 3.7. Lanthanum Oxide Nanoparticles

**Figure 7:**
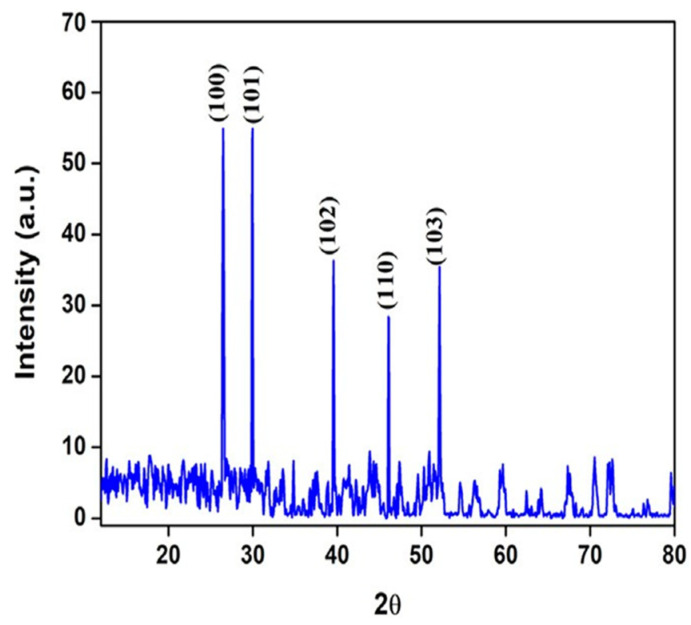
XRD pattern of La_2_O_3_. Reprinted with permission from Ref. [119]. Copyright 2021 Elsevier.

Recent research mostly focused on rare earth metal oxides (REOs) due to their extraordinary electrochemical and electrocatalytic characteristics and zero toxicity [117]. They have excellent structural stability, chemical, structural, and optical properties. The exceptional luminescence of REOs has promoted their use in environmental, technology, and industrial fields. Lanthanum oxide is recognized as a p-type material and has the highest dielectric constant, lowest lattice energy, and wide band gap (>4 eV), which makes it more desirable for a variety of biomedical and electronic applications [118]. Maheshwaran et al. [119] explored the biomedical applications of lanthanum oxide nanoparticles (La_2_O_3_ NPs) synthesized by utilizing the reducing potential of EG phytochemical and lanthanum nitrate. Before the preparation of NPs, EG extract was made by soaking EG leaves (20 g) in 50 mL of water and filtered through Whatman No. 1 filter paper. For La_2_O_3_ NPs fabrication, EG extract (10 mL) was mixed with 50 mL of 0.1 M solution of lanthanum nitrate, which was subsequently dried (60 °C) and annealed at 500 °C using a muffle furnace. XRD analysis (Figure 7) demonstrated the creation of hexagonal NPs with 52.21 nm average crystallite size. In the FTIR spectrum analysis, the La-O stretching band was found at 492.14, 630.4, and 866 cm^−1^, whereas the additional band represented the organic functionalities. The UV-Vis investigation revealed that La_2_O_3_ NPs have good optical characteristics with cut-off wavelengths of 290 nm. Lanthanum and oxygen were both visible in the EDX spectrum, with lanthanum making up around 81.6% and 18.4% of the total weight of the La_2_O_3_ NPs, respectively. The SEM images demonstrated the presence of particles with smooth surfaces but irregular particle morphologies that were positioned in multiple directions. TEM micrograph revealed smooth surfaced agglomerated La_2_O_3_ NPs having 50.37 nm average particle size. The intriguing results of La_2_O_3_ NPs as antioxidants, anti-diabetic agents, and anti-inflammatory agents indicated that La_2_O_3_ NPs may have future biological uses as alternative medications.

### 3.8. Gold Nanoparticles

The attributes of nanometric gold (Au NPs) vary exponentially from bulk gold due to the surface plasmon effect. At the macroscale, the bulk gold is considered biologically inert. The Au NPs have been used in biomedicine since the Middle Ages, when Arnald favored the use of potable gold, also referred to as aurum potabile, for medical purposes [120]. However, the medicinal applications of Au NPs were further explored and gained success after the advancement of nanotechnology [121,122]. The Au NPs produced using chemical procedure (reduction of chloroauric acid employing citrate solution) and a two-phase redox system (liquid-liquid) have various shortcomings, such as agglomeration, reduced stability of Au NPs as well as the hazardous effects on human health and environment [123]. Phytosynthesis has appeared as a viable alternative for the fabrication of stable and environmentally benign Au NPs of different morphologies [124]. Pinto et al. [125] opted for an eco-benign approach to synthesize Au NPs using an aqueous extract of EG bark and tetrachloroauric (III) acid trihydrate. The formation of Au NPs was performed by varying various experimental factors (pH, reaction time, and extract concentrations) to analyze the effect on the size and morphology. The pH study was carried out in acidic (2.7), neutral (7), and basic medium (10). A red shift (λ_max_ 525 nm→539 nm) was observed in the UV-Vis wavelength with the increase in pH of the solution, suggesting that the size of NPs has increased. Similarly, the DLS study of NPs synthesized at different pH showed an increase in hydrodynamic diameter with increasing pH. According to zeta potential measurements, Au NPs were found more stable at higher pH as compared to acidic pH. TEM analysis further supported these findings that an increase in pH led to the creation of larger size particles. The effect of extract concentration on particle size was observed by performing synthesis using different extract concentrations. A red shift in SPR peak (UV-Vis study), an increase in hydrodynamic diameter (DLS study), and an increase in particle size (TEM study) reinforced the results, and it can be deduced that a reduction in extract concentration led to the fabrication of larger size NPs. The temperature of the reaction has an important influence on the NPs synthesis. The impact of temperature on Au NPs synthesis was studied at 0 °C, 25 °C, and 80 °C. The zeta potential and hydrodynamic volume measured using DLS studies showed that temperature has a negligible effect on these parameters. UV-Vis studies showed SPR peak at nearly the same value, but an increase in intensity was observed. TEM investigation revealed a similar pattern, demonstrating that NPs produced at 25 °C and 80 °C had the same sizes. A further outcome of the TEM analysis was that the predominant morphology of the Au NPs produced under different sets of conditions was spherical in addition to prism-shaped structures.

Dzimitrowicz et al. [126] reported a comparative study on the Au NPs synthesis using EG and *Rosmarinus officinalis* essential oil and leaf extract. The following is a thorough explanation of Au NPs produced using EG extract, emphasizing the theme of the current investigation. UV-Vis results demonstrated the characteristic SPR peak of Au NPs synthesized using EG essential oil and leaf extract. Contrary to leaf-mediated Au NPs, EG oil-mediated Au NPs demonstrated two SPR peaks reflecting aggregated nanostructures in addition to a spherical. TEM analysis revealed that the average size of Au NPs produced employing leaves and oil extract was 12.8 and 42.2 nm with spherical and heterogeneous (hexagonal, spherical, rod-shaped, and triangular) morphologies, respectively. The EDX spectra revealed a strong Au signal, indicating that the precursor had been efficiently reduced in both cases, however additional signals of C, K, and O in the case of EG leaf-mediated synthesis were attributed to the availability of these components in the extract.

A comparative study on the fabrication of Au NPs was also carried out by Ibraheem et al. [127] using leaf extracts of EG, *Piper nigrum*, and *Ziziphus spinachristi*. The extract solution was prepared using a 1:10 extract-to-water ratio. The preparation of Au NPs utilizing EG extract was carried out using 10:1, 10:2, and 10:3 extract to metal ion precursor solution. UV spectra of Au NPs fabricated using EG extract showed a shift in SPR peak (λ_max_ 525→540 nm). XRD spectra showed an intense peak corresponding to 111 crystalline phase at all extract ratios, whereas a less intense peak corresponding to 200 phase was observed in the case of 2 and 3 mL of extract. The crystallite size of EG-mediated Au NPs was found to be 22 nm. SEM images showed an increase in particle size (28.6–32.1 nm) with an increase in extract ratio, demonstrating the accumulation of NPs with the increase in concentration.

### 3.9. Silver Nanoparticles

The synthesis of silver nanoparticles (Ag NPs) using EG bark broth as a capping/stabilizing agent and silver nitrate as a metal ion source was performed [128]. To make bark broth, bark samples (10 g) were finely ground and boiled in distilled water (100 mL) for 10 min. To prepare Ag NPs, 190 mL of salt solution and broth solution (10 mL) were mixed, then agitated for a day at ambient conditions in an incubator. UV-Vis spectra were recorded by taking a small fraction from the reaction medium to analyze the Ag NPs. SPR vibration of Ag NPs was observed at 440 nm with an increasing trend in absorbance with the increase in incubation time. This progressive rise in absorbance over time indicated increased Ag NPs generation. The average size of Ag NPs determined from XRD spectrum was found to be 30.5 nm. SEM images showed that synthesized Ag NPs possessed a wide range of morphologies, such as spherical, cubic, and hexagonal, having diameters in the 30–50 nm range. The EDX result exhibited an intense signal of silver (65.11%), confirming the presence of elemental silver in substantial amounts, along with faint indications for O, K, Mg, Cl, Al, and Si.

Ali and his co-worker explored the antibiofilm and antibacterial properties of silver Ag NPs prepared using EG leaf extract [129]. The preparation of Ag NPs was done by mixing EG extract and 1 mM silver nitrate solution, which was then irradiated with microwave for a 30 s period. After that, the mixture was allowed to cool to ambient temperature. For the optimization of reaction conditions, several reactions were carried out under different conditions by varying silver nitrate concentration (0.1–1 mM), extract concentration (1–5 mL), pH (4–1), incubation time (0–30 min), and temperature. UV-Vis, FTIR, SEM-EDX, XRD, TGA, and TEM investigations were carried out to analyze the morphological, structural, thermal, and optical aspects of Ag NPs. The Ag NPs were assessed for their antibacterial potential against *P. aeruginosa*, *E. coli*, MRSA, and MSSA. Ag nanoparticles showed an SPR peak at 428 nm. An increase in the intensity of Ag NPs absorption was observed with the increase in AgNO_3_ concentration, pH, EG extract, and reaction time, indicating enhanced production of Ag NPs at a higher value of these parameters. The XRD results showed the production of crystalline, face-centered cubic Ag NPs, whereas TEM images revealed variable and primarily spherical NPs having sizes between 5–25 nm and 1.9–4.3 nm synthesized with and without irradiation treatment. SEM results demonstrated the formation of spherical or oval morphology having smooth and irregular surfaces, while a feeble signal of silver along with O, C, K, Al, and Na in the EDX result revealed excessive capping of Ag NPs. The TGA plot indicated a consistent weight drop throughout a temperature range of 50–800 °C. The weight loss was attributed to the desorption of bioorganic components, indicating a robust interaction between EG extract and Ag NPs. The FTIR spectrum of EG-capped Ag NPs showed vibration bands that corresponded to numerous flavonoids, alkaloids, and other phytochemicals abundant in the extract. Antibacterial assay results indicated a dose-dependent action on microbial strains with an optimal concentration of 100 µL. The order of growth inhibition was found to be *E. coli* < *P. aeruginosa* ≈ MSSA ≈ MRSA. Gram-positive strains were shown to be more susceptible and vulnerable to Ag NPs than Gram-negative strains. In addition, significant biofilm inhibition was also seen for *S. aureus* and *P. aeruginosa* (83% and 84%, respectively).

Balamurugan and Saravanan [130] fabricated Ag NPs using EG extract and silver nitrate in different ratios. The absorbance of Ag NPs increased as the extract content, precursor salt concentration, and duration increased. Although the absorbance increased as the precursor concentration increased, however, the SPR peak was observed at nearly the same value. However, an increase in EG extracts up to 40 mL increase in absorbance was observed. Beyond this, a decrease in absorbance was seen along with a red shift in wavelength. Increased duration resulted in increased intensity and red-shifted absorption maximum, attributing to particle aggregation and increased size. XRD spectrum of Ag NPs fabricated using 20 mL of EG extract and 0.005 M silver nitrate solution was reported in the paper, which demonstrated the face-centered cubic Ag NPs. According to the TEM images, the majority of the particles were spherical, although some also had hexagonal and triangular morphologies with a size variation from 30 to 50 nm.

**Figure 8:**
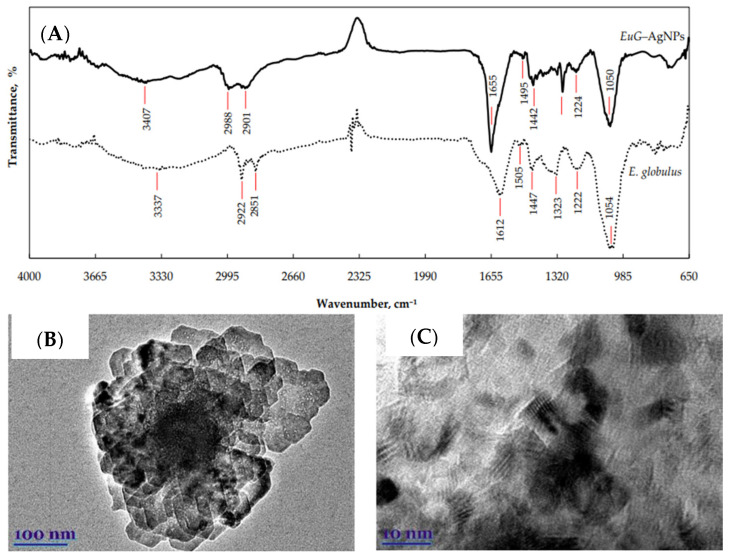
(**A**) FTIR spectrum and (**B**,**C**) TEM images of Ag NPs synthesized using EG leaf extract. Reprinted from Ref. [131].

BalCiUnaitiene and co-workers also adopted a greener approach for the fabrication of Ag NPs using EG leaves and *Salvia officinalis* leaf extract as a reducing agent and silver nitrate as a metal ions solution to investigate the biological utilities [131]. A comprehensive description is given below, keeping in its title. The Ag NPs were prepared by dissolving 30 mL of EG extract in 2.5 mL of AgNO_3_ while being stirred vigorously at ambient temperature for 2 h at a speed of 400 rpm. UV-Vis results were used to confirm the production of Ag NPs. The functional group characterization was carried out with FTIR analysis. Whereas morphological and elemental examination was done using TEM and EDX results. UV-Vis spectrum of EG-mediated Ag NPs exhibited an SPR peak at 408 nm, whereas the robust phytochemicals capping of Ag NPs was shown by peaks in the FTIR spectra that corresponded to different organic functionalities (Figure 8A). A morphological investigation by TEM revealed that the spherical shape predominated with an average diameter of 17.5 nm (Figure 8B,C). The antioxidant activity of EG-Ag NPs was estimated using DPPH, ABTS, and TFPH assays which showed that EG-capped Ag NPs demonstrated stronger antioxidant capability in comparison to EG extract. This enhanced activity was related to capped phytochemicals on the Ag NPs surface and the presence of silver in Ag^2+^ and Ag^+^ states. EG-Ag NPs and EG extract were tested for their antibacterial efficacy against both Gram-positive and Gram-negative bacteria. Gram-negativeve bacteria was found less susceptible to EG extract than Gram-positive bacteria. EG-Ag NPs exhibited potent antibacterial potential in comparison to extract with the zone of inhibition varying from 18.2–21.5 mm.

### 3.10. Titanium Dioxide Nanoparticles

Titanium dioxide (TiO_2_) normally exists in three solid states (anatase, brookite, and brookite) and possesses distinctive electrical, optical, magnetic, and thermal properties. TiO_2_ has extensive applications in orthodontic composites, paints, cosmetics, and food. The use of TiO_2_ NPs in photovoltaics, water splitting, photodegradation, sensing tools, electronic and electrochromic devices has sparked enormous interest and rapid progress in the manufacturing of TiO_2_ NPs [132,133,134,135]. Typically various chemical strategies are widely employed for the creation of TiO_2_ NPs. However, these approaches have restrictions because of the use of noxious reagents, drastic temperature, and pressure conditions. These shortcomings can be reduced by adopting a greener route (phytosynthesis) for the preparation of nanostructures [136,137,138]. Balaji et al. [139] explored the catalytic potential of TiO_2_ NPs fabricated using an environmentally benign approach. The fabrication of TiO_2_ NPs was carried out by dropwise addition of EG leaf extract as a reducing agent to 50 mL of ethanolic solution of titanium tetraisopropoxide while being vigorously stirred for 3 h. Centrifugation followed by washing, drying, and annealing resulted in nano titania synthesis. Different spectroscopic and analytical methods, including XRD, FTIR, TGA, SEM, DLS, and BET analysis, were carried out to confirm the composition of nano titania. Pristine anatase phase of nano titania, having an average particle size of 12 nm, was observed using XRD results. SEM and TEM images showed agglomerated spherical particles having a size in the range of 5–12 nm. In the FTIR spectrum, a less intense peak at 487.99 cm^−1^ reflected M-O bond formation, whereas feeble peaks corresponding to organic functionalities indicated their decomposition owing to high-temperature treatment. The TGA of nano titania revealed that the first substantial weight loss occurred at roughly 150 °C owing to water molecule evaporation, and the second major weight loss occurred between 150 and 340 °C due to the elimination of organic moieties. Synthesized nano titania had a BET-surface area of 50.94 m^2^/g and a 3.5 nm average pore diameter. TiO_2_ NPs were found as an efficient catalyst for pyran derivatives synthesis, which played its catalytic role by the activation of methylene and carbonyl group of malononitrile and aldehyde, respectively.

**Figure 9:**
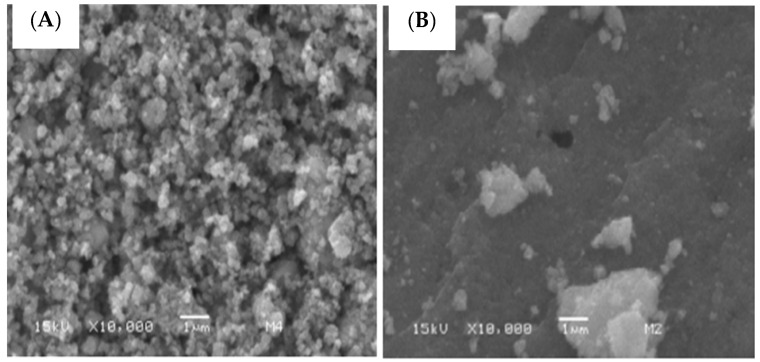
SEM micrographs of EG leaf extract mediated Ag-TiO_2_ aided by (**A**) microwave irradiation, (**B**) sol-gel method. Reprinted from Ref. [140].

Torres-LimiNana et al. [140] studied the comparative behavior of TiO_2,_ Ag-TiO_2_ NPs, and pristine Ag NPs as a water disinfectant synthesized using the green method (EG extract) and conventional method (sodium borohydride NaBH_4_). The EG extract was prepared using four different solvents (water, methanol, ethanol, and water/methanol), which were later used for NPs synthesis. Initial verification of Ag NPs fabrication was done using UV-Vis spectroscopy. Results showed characteristic peak of Ag NPs only for those synthesized using ethanolic extract. SEM images of Ag-TiO_2_ and TiO_2_ showed insignificant morphological differences synthesized using EG extract and sodium borohydride, whereas TEM images of TiO_2_ revealed spherical geometry. Similarly, for extract-mediated Ag-TiO_2_ NPs, spherical morphology having 11.4 nm was observed. SEM results revealed the spongy nature of EG extract-mediated Ag-TiO_2_ NPs irradiated by microwave compared to those prepared by using the sol-gel green method (Figure 9A,B). This was attributed to the external energy applied using microwave radiations. XRD and Raman spectroscopy results revealed the synthesis of TiO_2_ NPs in the anatase phase, whereas silver doping did not produce any change in crystallinity and band gap. TiO_2_, Ag, and Ag-TiO_2_ NPs were screened for antimicrobial potential against *S. aureus*. and *E. coli.* Microwave-aided EG and NaBH_4_ synthesized Ag and Ag-TiO_2_ NPs showed effective inhibition of both *S. aureus*_._ and *E. coli*. These results showed the potential utility of Ag and Ag-TiO_2_ as water disinfectants.

### 3.11. Zirconium Oxide Nanoparticles

**Figure 10:**
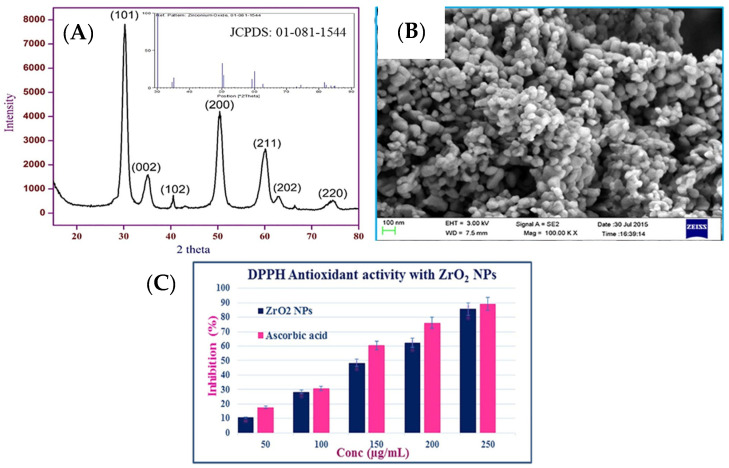
(**A**) XRD pattern and (**B**) SEM image of ZrO_2_ NPs, (**C**) comparative result of DPPH antioxidant activity of ZrO_2_. Reprinted with permission from Ref. [144]. Copyright 2017 Elsevier.

Phytosynthesis of zirconium oxide nanoparticles (ZrO_2_ NPs) is a one-pot robust, quick, facile, and environmentally safe approach since no noxious and hazardous chemical is utilized in the synthesis process [141]. ZrO_2_ NPs are extensively used as a coating in optical, ceramic, and dental industries as well as a catalyst in several organic reactions, piezoelectric and photocatalytic applications [142,143]. Balaji et al. [144] documented a maiden study on the biological evaluation of ZrO_2_ NPs fabricated using EG leaf extract. The synthesis comprised vigorously agitating a 0.1 N zirconium oxychloride solution with EG extract (50 mL). The resulting solution was centrifuged, washed, and dried in an oven at 80 °C. The dark-colored amorphous powder was eventually heated to 600 °C for three hours in a furnace to yield crystalline ZrO_2_ NPs. The ZrO_2_ NPs showed absorption maxima at 263 nm, whereas the UV-DRS results revealed a band gap of 3.15 eV. ZrO_2_ NPs displayed photoluminescence with a peak at 325 nm for excitation and 470 nm (400–580 nm range) for emission. XRD data revealed tetragonal phase ZrO_2_ having a 9.6 nm average size (Figure 10A). SEM results showed the formation of NPs having different morphology (rod-like and spherical (Figure 10B)).

The antioxidant and anticancer potential of ZrO_2_ NPs were assessed using DPPH and MTT assays. The ZrO_2_ demonstrated promising scavenging potential (85.6%), which can be related to reduced size (9.6 nm) and decrease in n→ π* transition intensity caused by electron density transfer from ZrO_2_ NPs to odd electron positioned at nitrogen atom in DPPH molecule (Figure 10C). ZrO_2_ NPs exhibited more toxicity to cancerous cells (lung and colon cancer cells) compared to healthy cells, which shows the potential of ZrO_2_ NPs to be used in the manufacturing of anticancer drugs.

## 4. The Probable Mechanism of Synthesis of Nanoparticles

**Figure 11:**
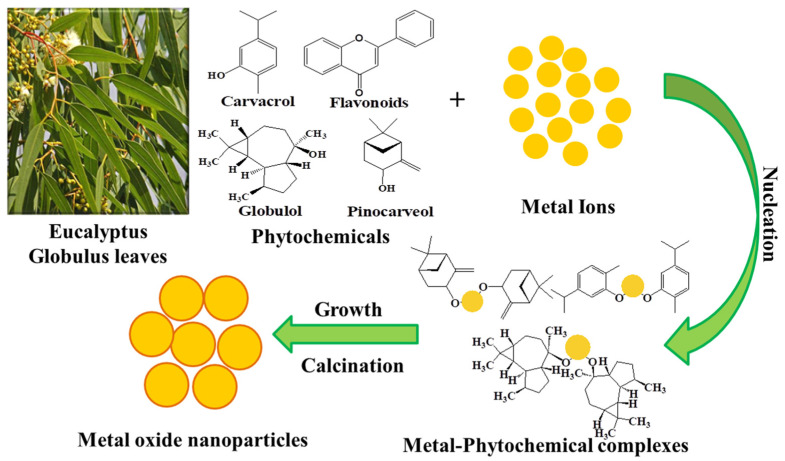
General mechanism of NPs synthesis using *Eucalyptus globulus* extract via bioreduction.

Plant extracts contain various phytochemicals, which are capable of synthesizing nanoparticles by functioning as capping and bio-reducing agents. The EG extract has an abundance of biomolecules, including proteins, flavonoids, alkaloids, carbohydrates, and phenols, which are liable for the bio-reduction and stabilization of metal salts into NPs. The hydrophobic phenolic constituents present in EG extract are supposed to attach to the metal salts and generate a metal complex. This metal complex will undergo an oxidation process resulting in the formation of nanoparticles. A detailed mechanism for the EG essential oil-mediated ZnO NPs was reported by Obeizi et al. [81]. Biphenolic compounds present in EG oil were supposed to play the role of reducing and stabilizing agents. The phytochemicals attached to the surface of ZnO NPs have extraordinary biological applications such as anticancer, antioxidant, bactericidal, and anti-diabetic potential [145,146]. The fabrication of Ag NPs through bioreduction using EG extract has been explained by BalCiUnaitiene et al. [131]. The bio-fabrication of Ag NPs was supposed to occur in three phases (activation, growth, and termination). During the activation phase, Ag^+^ ions were reduced and nucleated, whereas during the growth phase, adjacent Ag NPs aggregated to produce large particles, and during the termination phase, final morphology was formed. The general mechanism was supposed to be phytochelation, followed by bioreduction and growth process. A similar mechanism was also proposed for the fabrication of CuO NPs by Alhalili [93]. A general schematic of NPs fabrication utilizing EG extract is presented in Figure 11. In an aqueous solution, metal salts (most common metal salts are metal nitrates and metal acetates) are ionized to form metal ions (M^3+^, M^2+^, M^1+^), which are reduced to metal atoms (M^o^) by polyphenols (Carvacrol, globulol, pinocarveol) present in EG essential oils and extract. These polyphenols are hydrophobic and get attached to the surface of metal atoms to form metal-phytochemical complex/ metal-phenol complex M-OH). After heat treatment, M-OH is converted to metal oxide NPs. The NPs undergo aggregation during heat treatment [81,93]. The synthesis of metallic NPs from EG can be divided into three stages, (1) activation phase, during which reduction and nucleation of metal ions occur, (2) growth phase, during this phase coalescence of neighboring metal NPs occur to form larger particles, which result in the increased thermodynamic stability of NPs, and (3) termination phase, resulting in the final shape of the NPs [131].

## 5. Advantages and Challenges of Green Synthesis

The green methodologies of NPs synthesis have emerged as preferred alternatives to the chemical and physical procedures owing to their simple, clean, and environment friendliness nature. Green synthesis is recognized as an ecological and cost-convenient route since it makes use of easily accessible biological sources such as fungi, algae, plants, and microorganisms which act as bioreductant and stabilizing/capping agents [147]. Additionally, there is no requirement for advanced instrumentations, high temperature and pressure compulsions, and hazardous chemicals for the preparation of NPs [148]. Furthermore, green methods do not require any sophisticated purification technique, do not produce any hazardous byproducts, and the use of water (a universally recognized biocompatible solvent) as a reaction medium further support the use of these methods [149,150]. Phytochemical-mediated process of NPs synthesis has emerged as a preferred option among all the green methods (mycological, bacteriological, and phycological approaches) for the fabrication of stable NPs. The preference for plant-assisted synthesis over other methods can be justified by the advantages of utilizing plant extracts, such as the variety of metabolites, ease of availability, and easy and faster synthesis of NPs that are environmentally benign and free of adverse effects. Furthermore, plant-based synthesis is not tedious since it does not necessitate the production and nurturing of cell cultures [151,152,153].

**Figure 12:**
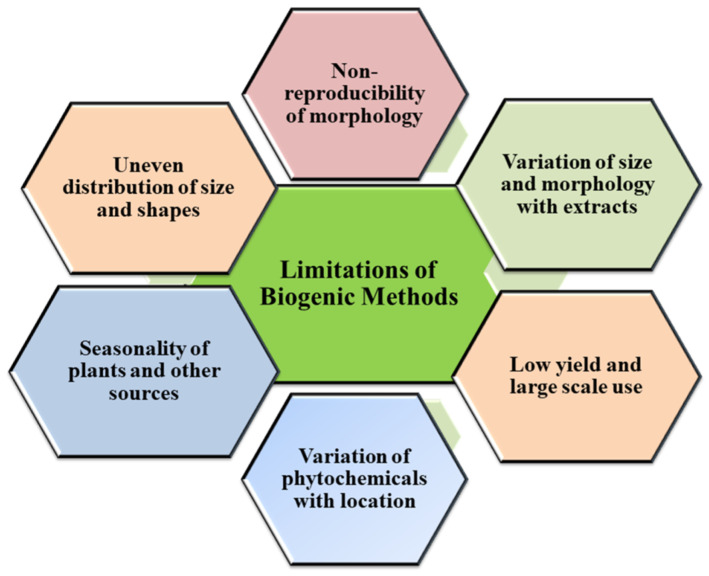
Drawbacks of biogenic methods.

Although the green methods of NPs synthesis using biological sources are appealing alternatives to toxic chemical strategies, however, there are a few limitations associated with these methods. The main drawback of the green method is the difficulty in effectively controlling the particle morphology, size distribution, and the production of reproducible morphology. The morphology/shape and size of NPs fabricated using different biogenic sources differ greatly. Similarly, the phytochemical makeup of plants varies with their locations, which leads to the variation of morphology and size of NPs fabricated in different laboratories. One of the key challenges linked with biogenic methods is the scaling up of the synthesis method. Furthermore, the precise mechanisms of NPs production employing biological methods have not yet been clarified [154,155]. The major shortcomings of green synthesis are presented in Figure 12.

In order to overcome the above-mentioned limitations and to put phytosynthesized nanomaterials to real-world applications, the following suggestions can make green methods more fruitful. The problem related to seasonality and phytochemical variation can be resolved by considering the seasonal and regional availability of plants. Plants that are not limited to a specific area and season should be explored in the future for the synthesis of NPs. For example, plants belonging to the Pine family have been explored for the synthesis of NPs [77,156,157]. These readily available evergreen plants could avoid the limitation of seasonal availability and can be employed as an alternative to seasonal plants. The extract prepared either using leaves collected from different trees of the same plant or under different conditions (temperature, using different amounts of plant materials, etc.) might result in the variation of morphology and size of nanoparticles. This can be avoided by using the extract prepared under the same experimental conditions and using materials collected from the same plant. Sawaha et al. synthesized Au NPs having uniform spherical morphology and 68.44 nm size using *Hygrophila spinosa* [158]. Similarly, Naika et al. fabricated CuO NPs having a uniform particle size in the range of 5 to 10 nm using an extract of *Gloriosa superba.* The limitation of uniform particle size and morphology can be overcome by exploring the phytochemical makeup of plants and selecting the appropriate one that can produce NPs of desired size and morphology. Similarly, variation in plant extract to metal salt solution ratio results in the generation of NPs of different sizes and morphology. Thus, NPs of the desired size and morphology having even distribution can be fabricated by varying extract to salt ratio and then selecting the one that produces the desired results. Despite the minor drawbacks, the benefits demonstrated by these green methods are significantly greater, which favors their widespread usage for the preparation of a variety of nanostructures [149,150,151,152,153,154].

## 6. Conclusions and Future Prospective

In summary, the present review entailed a description of the role of EG in the phytosynthesis of nanostructures as a promising alternative route to the conventional methods for the non-toxic, eco-benign, and low-cost production of nanoparticles. An attempt is being made to explain the synthesis route, characterization results, and studied applications of different NPs produced using EG leaves and bark extract, as well as essential oils. EG plant contains a variety of phytochemicals that serve as bioreductant and capping agents for the fabrication and stabilization of NPs. The synthesis of NPs of various metals having different morphologies and sizes depicts the potential of EG phytochemicals to be utilized as potential stabilizing and reducing agents. The majority of the synthesized nanoparticles have demonstrated their usefulness in different scientific fields, such as photocatalysis and biomedical domains.

Future prospects of EG include the fabrication of doped nanomaterials, nanocomposites, and high entropy oxides of various other metal salts for biomedical, environmental, and energy applications. Considering the immense significance of EG extract-mediated NPs as photocatalysts, antibacterial and antioxidant agents, it is expected that EG phytosynthesized NPs will eventually be beneficial in environment remediation and biomedical applications. Similarly, the anticancer activity of EG phytochemicals has been explored, therefore, it can be anticipated that NPs synthesized using EG extract can also be used as theranostic agents in cancer therapy. Although the hypothetical mechanism involved in the phytosynthesis of nanomaterials has been proposed by various groups, future studies must emphasize the actual process underlying the mechanism of synthesis.

## Figures and Tables

**Table 1:** The pharmacological activities of different parts of EG plants are given below.

Pharmacological Activity	Method Used	Plant Parts	Reference
Antibacterial	Agar diffusion method, Disc diffusion method	Leaves, ariel part, fruit	[35,39,52,53,54]
Anticancer	MMT assay	Fruit, essential oils	[55,56]
Antioxidant	DPPH assay, ABTS method	Leaves	[39,57,58,59,60]
Antifungal	Broth microdilution assay, double dilution micro-plate assay, disc diffusion method	Leaves	[49,61,62,63,64]
Neuro-protective	MMT assay	Leaves	[57,65]
Anti-hyperglycemic	Monitored oral glucose tolerance test	Leaves	[66]

**Table 2:** A comparison of morphology and size of ZnO NPs phytosynthesized using EG extract.

Plant Parts	Significance	Morphology	Size (nm)	Reference
Leaves	Photocatalytic and antioxidant activities	Spherical and hexagonal	10–20	[74]
Leaves	Antibacterial, antioxidant, anticancer activities	Spherical	10–30	[78]
Leaves	--	Hexagonal	35	[79]
Leaves	Antifungal activity	Agglomerated clusters	52–70	[80]
Essential oils	Antimicrobial and anti-biofilm activities	Spherical and needle-like	40	[81]
Leaves	Photocatalytic activity	Spherical	--	[82]
Leaves	Antibacterial activity	--	--	[83]
Leaves	Antimicrobial activity	Spherical, flower, and walnut shape	186.7	[84]
Leaves	Photocatalytic activity	Agglomerated shape	--	[85]

## Abbreviations

ABTS	2,2’-azino-bis(3-ethylbenzothiazoline-6-sulfonic acid)
Ag	Silver
Au	Gold
*A. mali*	*Alternaria mali*
*B. dothidea*	*Botryosphaeria dothidea*
CuO	Copper oxide
DLS	Dynamic light scattering
DSC	Differential scanning calorimetry
*D. seriata*	*Diplodia seriata*
DPPH	2,2-diphenyl-1-picrylhydrazyl
*E. coli*	*Escherichia coli*
EDX	Energy-dispersive X-ray spectroscopy
EG	*Eucalyptus globulus*
EOs	Essential oils
FTIR	Fourier transform infrared spectroscopy
*K. leb*	*Klebsiella pneumonia*
La_2_O_3_	Lanthanum oxide
MSSA	Methicillin-sensitive *Staphylococcus aureus*
MRSA	Methicillin-resistant *Staphylococcus aureus*
*MTT*	3-(4,5-Dimethylthiazol-2-yl)-2,5-diphenyltetrazolium bromide
NPs	Nanoparticles
*P. aeruginosa*	*Pseudomonas aeruginosa*
PbO	Lead oxide
SEM	Scanning electron microscopy
SPR	Surface plasmon resonance
TEM	Transmission electron microscopy
TFPH	Trifluoperazine dihydrochloride
TGA	Thermogravimetric analysis
TiO_2_	Titanium dioxide
XPS	X-ray photoelectron spectroscopy
ZnO	Zinc oxide
ZrO_2_	Zirconium oxide
